# dic(7;9)(p11–13;p11)

**DOI:** 10.4267/2042/70583

**Published:** 2019-04

**Authors:** Mary J Underdown, Thomas B Russell, Mark J Pettenati, David E Kram

**Affiliations:** Department of Pediatrics, Wake Forest School of Medicine, Winston Salem, North Carolina (MJU); Section of Pediatric Hematology-Oncology, Department of Pediatrics, Wake Forest School of Medicine, Winston Salem, North Carolina (DEK, TBR); Department of Pathology, Wake Forest School of Medicine, Winston Salem, USA (MJP), Medical Center Boulevard, Winston Salem, NC, USA 27157;

**Keywords:** Acute lymphoblastic leukemia, dicentric translocation, PAX5, chromosome 7, chromosome 9

## Abstract

Dicentric (7;9)(p11–13;p11) is a rare but recurrent abnormality in pediatric and adult precursor B acute lymphoblastic leukemia (B-ALL). The rarity precludes a deep understanding of its biology and associated prognosis. However, recent findings have correlated dic(7;9) and PAX5 mutations, highlighting this cytogenetic event’s involvement in leukemogenesis and may also shed light on the overall prognosis of dic(7;9) B-ALL.

## Clinics and pathology

### Disease

ALL

### Phenotype/cell stem origin

FAB L1 phenotype; pre-B immunophenotype, cIg+ or cIg−

### Epidemiology

There have been 36 cases of dic(7;9)(p11–13;p11) currently identified in the literature, 17 (47.2%) of which are pediatric cases. This rare translocation makes up < 1% of childhood ALL, It is most commonly found in younger children, age ≤ 6 years; dic(7;9)(p11–13;p11) is found in approximately 3% of childhood ALL with 9p abnormalities and has been associated with B-ALL with t(9;22), or Philadelphia chromosome positive ALL.

### Clinics

The most common clinical manifestations of dic (7;9) noted in the literature include age female, T- and BALL with B-cell predominance, leukocytosis 9, enlargement of liver/spleen/lymph nodes (Pan and Xue, 2006).

### Prognosis

Favorable prognostic indicators in ALL include: age 1–10 years, female sex, Caucasian or Asian ethnicity, WBC count <50,000 at presentation, B-cell immunophenotype, hyperdiploidy, and trisomy of chromosome 4 or 10. The most important prognostic factor is end of induction therapy minimal residual disease (MRD) (Hunger and Mullighan, 2015; Iacobucci and Mullighan, 2017).

However, abnormalities in chromosome 9p or deletions of the tumor suppressor genes located on 7p have been associated with increased rates of relapse (Jarosova and Volejnikova, 2016), and may even potentially trump favorable NCI criteria or other favorable cytogenetics.

## Cytogenetics

### Note

Several dicentric chromosomes found in childhood ALL are formed from the q arms of chromosomes 7, 9, 12, and, 17 with partial loss of the respective p arms.

### Cytogenetics morphological

Unbalanced; In most cases, formation of a dicentric chromosome resulting in partial monosomies of 7p and 9p -> hypodiploid with 45 chromosomes. However, hyperdiploidy (56 chromosomes) has been identified.

### Additional anomalies

del(6q), dup(1p), del(8p),…

## Genes involved and proteins

### PAX5

**Location** 9p13.2

### Note

Recent studies have shown an association between dic(7;9) and PAX5 mutation. PAX5 encodes the B lymphoid transcription factor gene and is vitally important in regulating B cell lineage differentiation. PAX5 alterations may lead to arrested B-cell development in the pro-B-cell stage and may be central events in B lymphoid leukemogenesis (Shah and Schrader, 2013).

A recent study by Bastian, et. al. found 19/250 pediatric and adult patients with B-cell precursor ALL harbored PAX5 mutations. Of these patients with PAX5 mutations, 12/19 (63%) had alterations in chromosome 9, though the specific cytogenetic alterations were not reported (Bastian and Schroeder, 2019).

A large cohort study out of St. Jude’s identified 17/1988 (0.86%) patients with dic(7;9) translocation; of those, 11/17 (65%) had a PAX5 alteration or mutation. While the PAX5 gene is located on 9p13.2, 5/11 (45%) cases with dic(7;9)(p11;p11) were associated with PAX5 alterations. This study found two distinct subtypes of B-ALL characterized by PAX5 alterations: the first (n=148) which harbor diverse PAX5 alterations (including rearrangements, sequence mutations, and focal intragenic amplifications) and the second (n=44) which harbor a particular nonsilent sequence mutation, PAX5 p.Pro80Arg. As a group of all PAX5, the 5-year event-free survival was variable, ranging from 50% to 75% (Gu and Churchman, 2019).

## Figures and Tables

**Figure 1:**
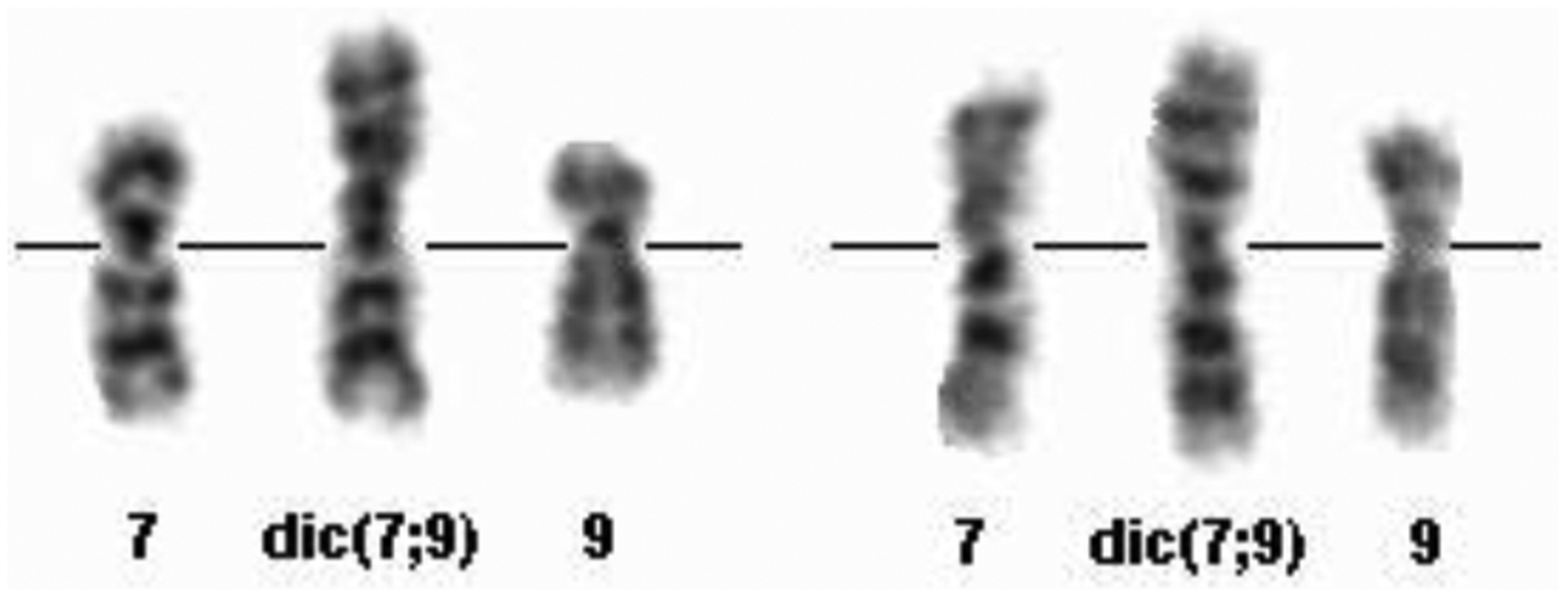
dic(7;9)(p11–13;p11) G-banding - Courtesy Cytogenetics Laboratory of the CCRI, Children’s Cancer Research Institute, Vienna.

**Figure 2:**
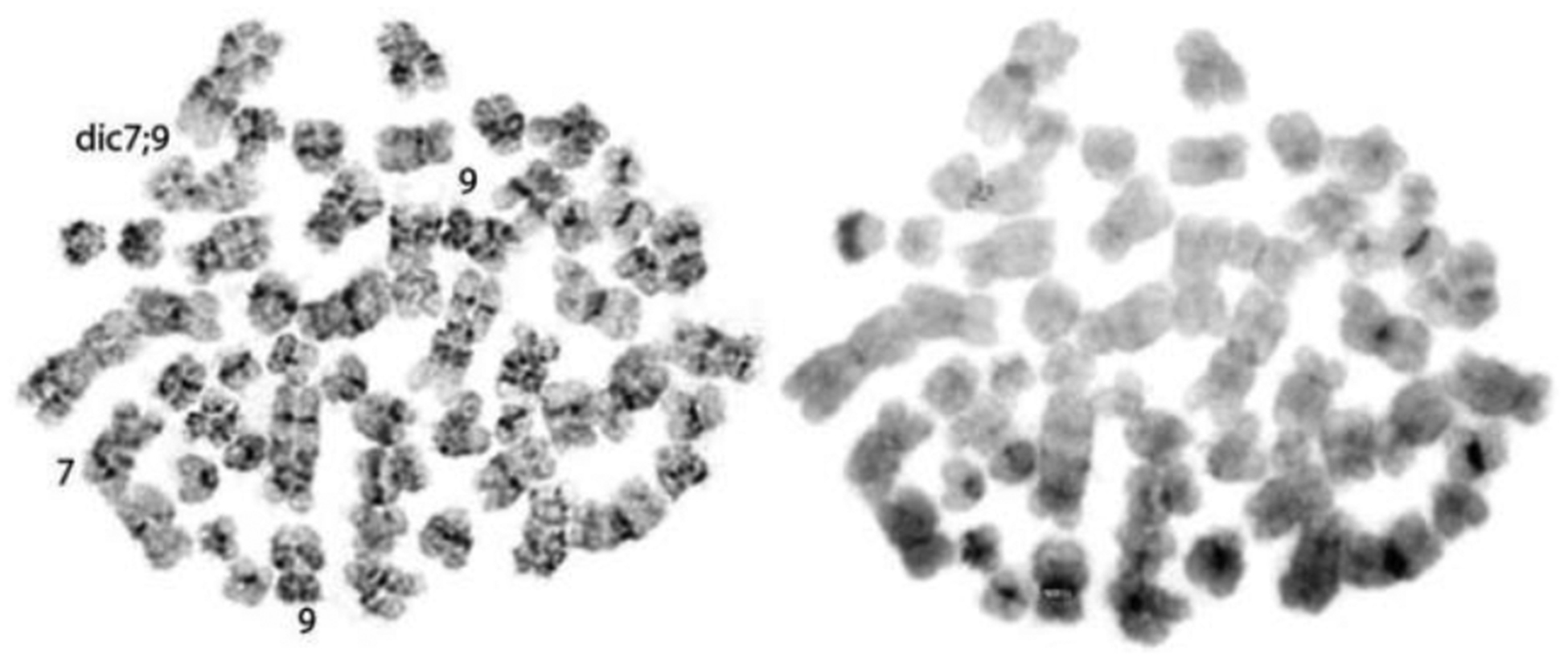
FISH image depicting 7; dic 7–9; 9 with centromeric probes of 7 and 9 fused - Courtesy Department of Pathology, Wake Forest School of Medicine, Winston Salem, USA.
